# Lipofibromatous Hamartoma of the Superficial Peroneal Nerve with Significant Macrodactyly and Macrodystrophia Lipomatosa

**DOI:** 10.1055/s-0044-1793819

**Published:** 2024-11-19

**Authors:** Rohini Prasad, Arun Kumar Rajeswaran, Karishmah Senthil Kumar, Purvashree Prasad

**Affiliations:** 1 Department of Plastic Surgery, Avinash Hospitals, Chennai, Tamil Nadu, India; 2 Department of Plastic Surgery, Apollo Hospitals, Chennai, Tamil Nadu, India; 3 Department of Plastic Surgery, Gleanagles Global Hospital, Chennai, Tamil Nadu, India; 4 Velammal Medical College Hospital and Research Institute, Chennai, Tamil Nadu, India

**Keywords:** lipofibromatous hamartoma, macrodystrophia lipomatosa, superficial peroneal nerve

## Abstract

We report a rare presentation of lipofibromatous hamartoma involving the superficial peroneal nerve with significant macrodactyly in a woman in her late 40s, with a follow-up period of 3 years. We discuss the clinical presentation, distinguishing features, and the surgical technique chosen for maximum functional outcome, along with a review of the literature. This case report attempts to understand the spectrum of disease represented by lipofibromatous hamartoma, its nomenclature, diagnosis, and management depending on the extent of tissue involvement. In this case, as there was macrodystrophia with osseous overgrowth and extensive lipomatosis, the challenges involved in planning surgery for a desirable aesthetic and mechanical outcome have been discussed.

## Introduction

Lipofibromatous hamartoma (LFH), intraneural lipoma, perineural lipoma, and neural fibrolipoma are subsets of hamartomas that typically present as fibroadipose soft tissue masses within the epineurium of a nerve. 1 It is a benign tumor of nervous tissue that most commonly involves the upper limb, more specifically the median nerve 2 3 associated with pain and sensory and/or motor deficits in the area of innervation of the affected nerve. There have been very few documented cases of LFH involving the foot, and among these cases, growth of bone and other tissue causing macrodactyly, a condition known as macrodystrophia lipomatosa (MDL), is observed in less than 25% of cases. We describe the diagnostic process, surgical resection, and reconstructive techniques in an atypical presentation of a large, long-standing LFH of the foot with significant deformity and the follow-up results 3 years after surgery.

## Case Report

The patient, in her late 40s with no known comorbidities, presented to the outpatient clinic with a deformed left foot and multiple bulbous lesions present over the dorsal and plantar aspects. The swelling first appeared at the age of 10 years and progressively increased over three decades. As the tumor caused gigantism of the bone and macrodactyly with loss of normal foot anatomy, the patient presented with pain on walking and difficulty in gripping the footwear.

**Fig. 1:**
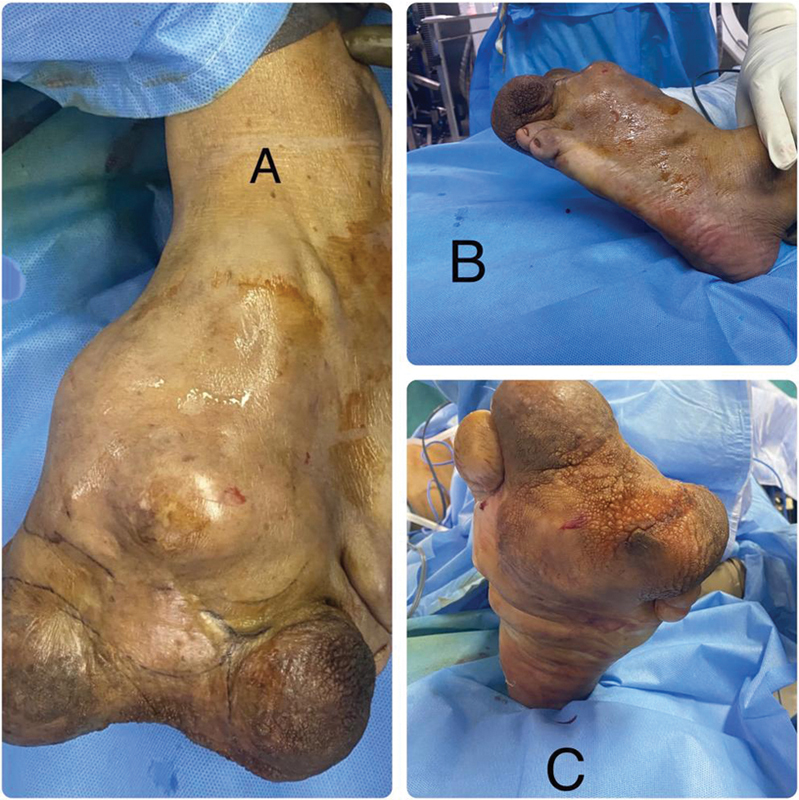
Preoperative appearance of the macrodactylous foot with multiple lumps. ( **A** ) Dorsal view. ( **B** ) Lateral view. ( **C** ) Plantar aspect.

The masses were firm and slightly mobile, and tender with negative transillumination. The left first, fourth, and fifth toes were identifiable but grossly compressed. The remainder of the foot was completely disfigured and distorted, with the tumor infiltrating both the dorsal and plantar aspects of the foot, extending up to the ankle joint ( Fig. 1 ).

**Fig. 2:**
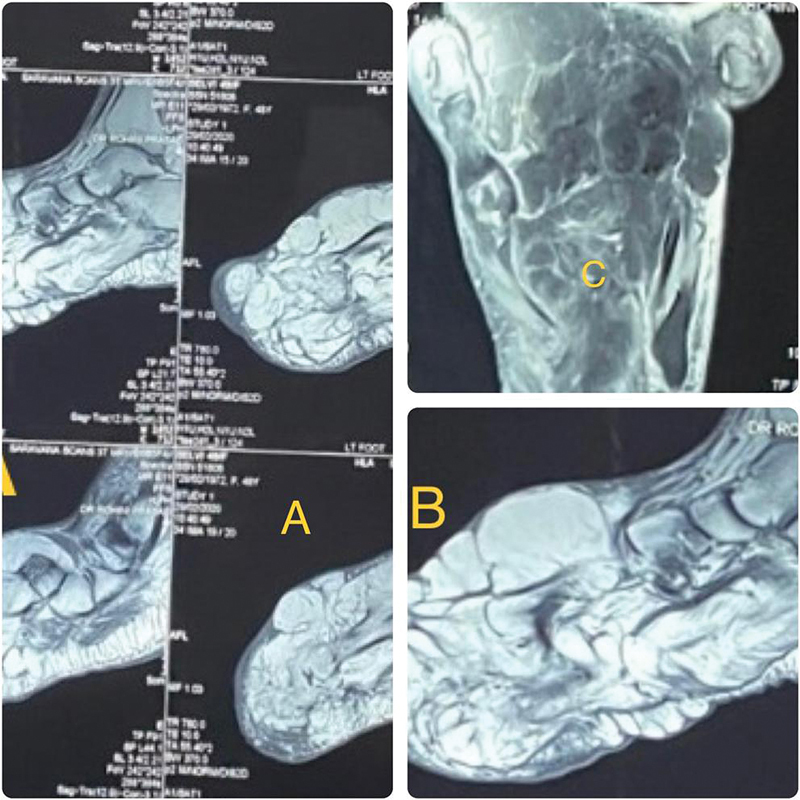
( **A-C** ) Magnetic resonance imaging showing showed hypointense nerve fascicles of the superficial peroneal nerve, surrounded by multiple encapsulated hyperintense fibroadipose tissue.

No sensory or motor deficits were noted and nerve conduction study was normal. Magnetic resonance imaging (MRI) of the left foot ( Fig. 2 ) showed hypointense nerve fascicles of the superficial peroneal nerve (SPN), surrounded by multiple encapsulated hyperintense fibroadipose tissue, clinically referred to as MDL. 4

## Procedure

In view of the recent rapid progression, surgical treatment was planned, which involved resection of the tumor along with nerve and bony mass and reconstruction of the defect.

**Fig. 3:**
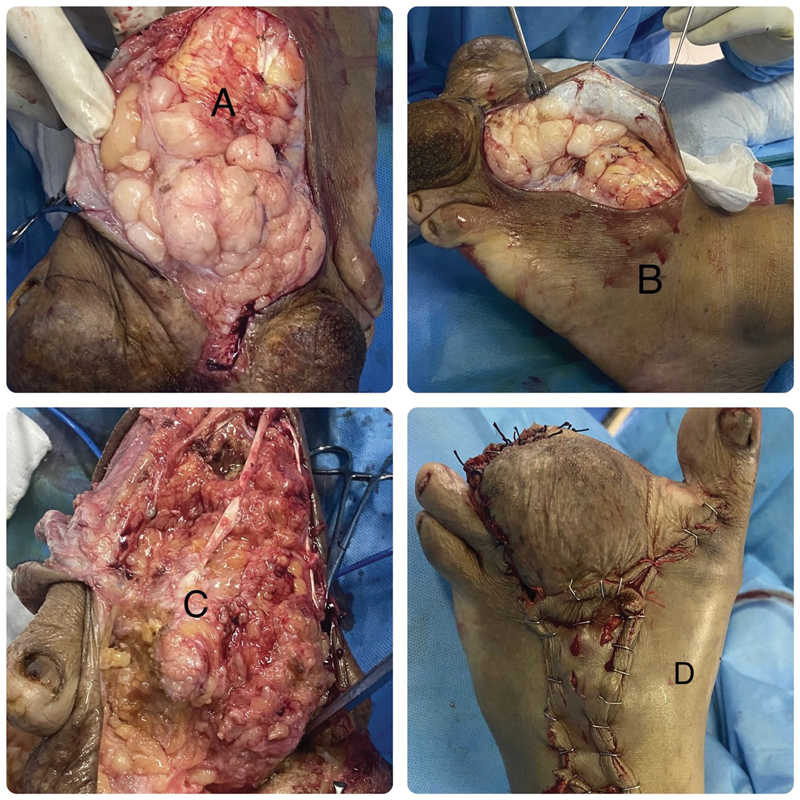
Intraoperative images. ( **A** ) Exposure of the tumor, ( **B** ) excision of the lesion, ( **C** ) disarticulation at the second and third metatarsophalangeal joints, and ( **D** ) reconstruction with plantar skin flaps and split skin graft to cover the metatarsal heads and dorsal raw area.

Intraoperatively, the second and third left toes exhibited macrodactyly with no identifiable anatomy. The dystrophic bony mass corresponding to the second and third phalanges was resected, the second and third metatarsophalangeal joints were disarticulated and the metatarsal heads were partially excised. The medial dorsal cutaneous branch (MDCB) of the SPN was hypertrophied from a region just distal to the anterior annular ligament. The tumor had a thin capsule with multiple septa and the branch was completely involved by the tumor; however, it had displaced the lateral branch up to the lateral border of the foot, and the deep peroneal neurovascular bundles were intact but flattened deep to the tumor. The plantar masses, which were multiple, encapsulated, and compartmentalized, were excised through separate incisions and none of the plantar nerves were involved. The foot was reconstructed with plantar and dorsal flaps, which were raised to reach over and cover the lipomatous tumor cavity and the bony hypertrophy. The plantar flap was used to cover the webspace between the first and fourth toes, and the residual raw area over the dorsum was covered by a graft harvested from excised skin ( Fig. 3 ).

**Fig. 4:**
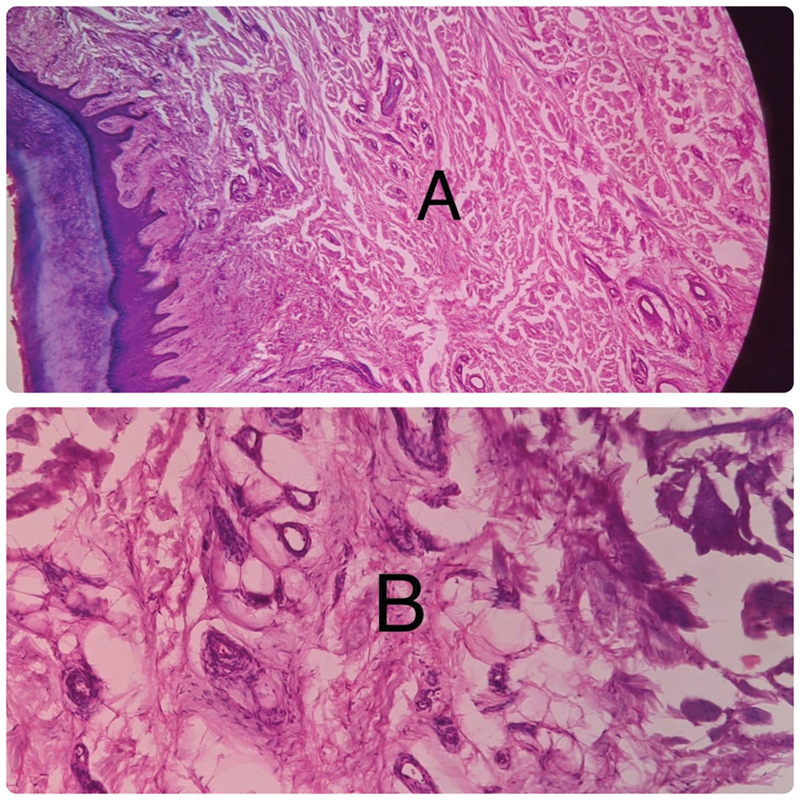
Photomicrograph ( **A** ) showing stratified squamous epithelium with subepithelium ( **B** ) showing fibrocollagenous stroma enclosing lobules of mature adipocytes separated by fibrous septa.

The histopathology report revealed stratified squamous epithelium with the subepithelium showing fibrocollagenous stroma enclosing lobules of mature adipocytes separated by thin and thick fibrous septa, as enclosed in figure confirming the diagnosis of LFH ( Fig. 4 ).

**Fig. 5:**
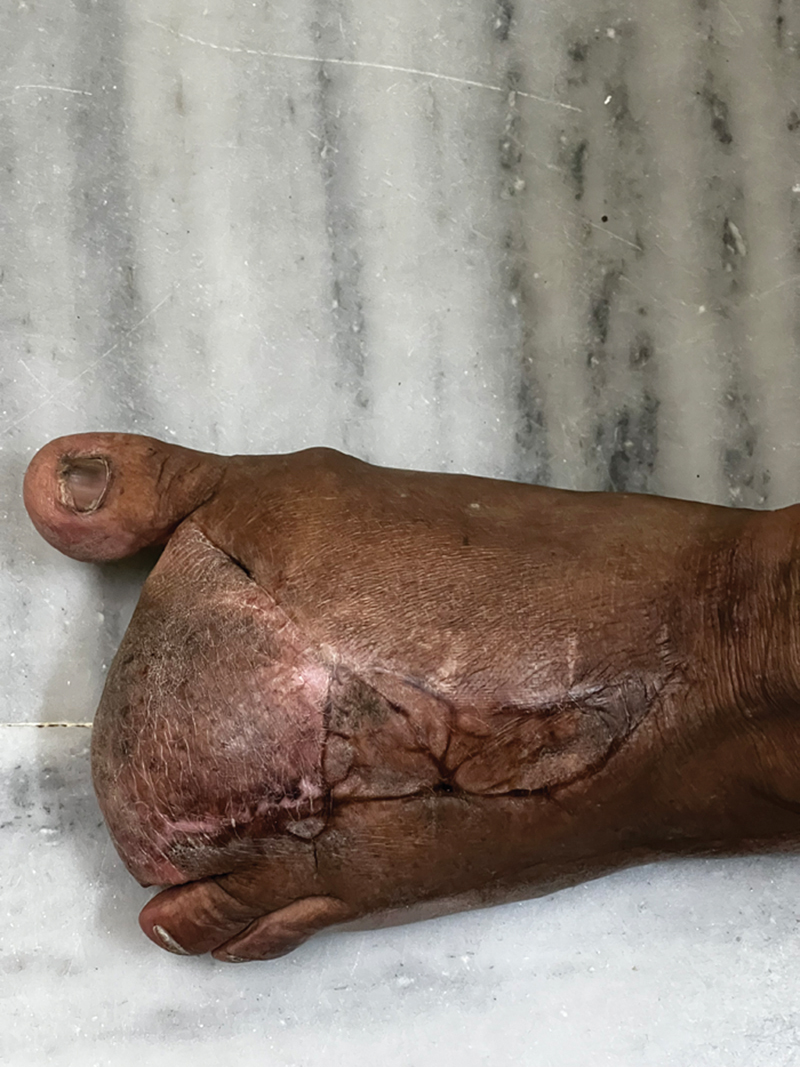
Two-year follow-up picture of the foot with full weight bearing.

Postoperatively, all wounds healed by the fifth week and she was started on compression garments, customized footwear, and full weight bearing. During her 3-year follow-up, it is noted that she could walk comfortably and wear her footwear with no difficulties, with no recurrence ( Fig. 5 ).

## Discussion

LFH is an anomalous growth of fibroadipose tissue of the peripheral nerve sheath occurring usually before the third decade sometimes associated with macrodactyly. The commonest nerve to be affected is the median nerve sometimes causing carpal tunnel syndrome. 4

LFH with macrodactyly appears to be more common in women, 1 but when it is not associated with macrodactyly, both males and females seem to be equally affected. LFH is the most common condition associated with macrodactyly in the hand but is only rarely associated with macrodactyly in the foot. Amadio et al described 17 cases of LFH of the nerve, of which 4 cases were in the lower extremity: 3 involving the medial plantar nerve and 1 involving the ankle. 5 Silverman and Enzinger described 26 cases of LFH of the nerve, of which 25 involved the upper extremity. 1 Cases of LFH involving the tibial nerve and sciatic nerve have been documented. 6 7

MDL is a distinct clinical entity coined by Feriz in 1925 to describe lower extremity overgrowth commonly occurring in children due to lipomatosis. 8 It causes localized gigantism and disproportionate growth, leading to difficulty in both ambulation and the use of normal footwear as noted in our patient.

No causal link has been established between hamartomas/pathological involvement of a nerve and soft tissue enlargement. LFH of peripheral nerves can be associated with MDL but can even occur without macrodactyly. True macrodactyly is characterized by diffuse enlargement of all the structures such as nerves, bone, vessels, and subcutaneous fibrous fatty tissue, as evidenced in our patient. 9 MDL is associated with a specific sclerotome 10 region of the body. The fibrofatty tissue and bone (second and third rays) associated with hypertrophy, exostosis, and ankylosis of the interphalangeal (IP) joints are all localized to the sclerotome represented by the MDCB of the SPN, while the overgrowth displaces the rest of the normal tissue.

There are around seven to eight described cases of LFH involving the SPN and only one of them has been associated with macrodystrophia. The rest presented as nodular growths, which could be easily resected and soft tissue closure obtained. 11 We present a case of LFH of the entire foot with macrodystrophia, involving the SPN for which extensive resection and reconstruction was attempted successfully.

Surgical management of LFH is planned for functional impairment to the limb (as in our patient), extensive pain and deformity, loss of sensation in the limb, unsightly appearance of the tumor affecting the patient's psychosocial state, and sudden increase in the size and shape of the tumor.

The aims of the surgery were to avoid amputating the foot, retain sufficient residual metatarsal head for appropriate weight bearing postoperatively, preserve the intrinsic muscles to give the foot good support, preserve the medial and lateral plantar nerves, and reconstruct the plantar sole so it is soft and pliable and not prone to ulceration or callosities.

There were many challenges during planning and execution of the surgery as we could not assess the true size of the defect. It was not possible to anticipate the size of the flap required, but raising thicker flaps ( **U** and **C** shaped) for resection helped resolve this. The space created after disarticulation of the second and third metatarsal heads was then used to create a webspace between the first and fourth toes with a plantar fillet flap. This was important to maintain the mediolateral width of the forefoot. Closure of the residual defect of the dorsum using graft harvested from excised skin as the defect was not small enough to close primarily and did not warrant a flap.

## Conclusion

LFH occurring in the lower extremity is uncommon and those involving the SPN are extremely rare. The current nomenclature based on involved nerve does not help in describing the extent of involvement. LFH is a spectrum of disease that can be diagnosed by MRI and confirmed by histopathology. The treatment of choice for LFH with MDL with extensive architectural distortion is radical excision of the tumor, followed by meticulous reconstruction, to achieve a superior mechanical and aesthetic outcome.
